# Use of Network Pharmacology to Investigate the Mechanism of the Compound Xuanju Capsule in the Treatment of Rheumatoid Arthritis

**DOI:** 10.1155/2021/5568791

**Published:** 2021-08-10

**Authors:** Wenyang Wei, Wanpeng Lu, Xiaolong Chen, Yunfeng Yang, Mengkai Zheng

**Affiliations:** Academic Research and Development Center of Zhejiang Strong Pharmaceutical Co., Ltd., Hangzhou, 310053 Zhejiang, China

## Abstract

**Objective:**

To clarify the therapeutic mechanisms of compound Xuanju capsule-treated rheumatoid arthritis (RA) based on network pharmacology tactics.

**Method:**

The TCMSP, TCMID and STITCH databases were used to screen the active ingredients and targets in the compound Xuanju capsule; the OMIM, TTD, PharmGKB and GeneCards databases were applied to screen the RA-related disease targets. Then, the obtained targets were imported into Cytoscape 3.7.1 software to construct the active ingredient-target network and the RA-related disease-target network. The active ingredient-target PPI network, the RA-related disease-target PPI network and the common target PPI network were built by using the STRING platform and Cytoscape 3.7.1 software. The GO and KEGG analyses of the common targets were analyzed by using the Metascape and Bioinformatics online tools.

**Results:**

A total of 51 active ingredients and 513 corresponding ingredient targets were harvested from the compound Xuanju capsule; 641 RA-related disease targets were obtained. After two PPI networks were constructed and merged, 116 RA-related targets of compound Xuanju capsules were identified and analyzed. 116 RA-related targets of compound Xuanju capsules are mainly involved in the biological processes and molecular functions, such as the cytokine-mediated signaling pathways, the response to lipopolysaccharide and the blood vascular development, the cytokine activity, the cytokine receptor binding and the receptor regulator activity. Furthermore, 116 RA-related targets of compound Xuanju capsules are concentrated in signaling pathways such as the IL-17, TNF, Th17 cell differentiation, Toll receptor and RA signaling pathway.

**Conclusion:**

The compound Xuanju capsule had the action characteristics of multiple components, multiple targets, and multiple pathways in the treatment of RA, which might primarily reduce the release of proinflammatory factors (such as IL-6 and TNF-*α*) and increase the production of anti-inflammatory factors (such as IL-10) by regulating inflammation-related signaling pathways (such as IL-17), thereby alleviating the inflammatory damage and improving the bone tissue repair.

## 1. Introduction

Rheumatoid arthritis (RA), a common autoimmune dysfunction disease characterized by the chronic inflammation of the joint synovium and the systemic vasculitis, can severely impair the physical function and the quality of life [1]. Worldwide, approximately 1% of the population is affected by RA, and the RA prevalence in women is about three times higher than that in men [2]. The pathogenesis of RA is relatively complicated and related to immune imbalance, genetic factors, environment factors, and so on, in which the participation of cytokines plays an important role [3, 4]. The common therapeutic drugs for RA include the nonsteroidal anti-inflammatory drugs, the disease-modifying antirheumatic drugs, glucocorticoids and biologic agents. While these drugs are taken, there are also the long-term side effects such as increased risk of infection and potential fatal liver damage [5]. Traditional Chinese Medicine (TCM) has been used to treat RA for more than three thousands of years in China. Modern pharmacological researches demonstrated that the effective active ingredients in Chinese medicine could weaken the activity of RA and protect joints from deformity [6]. In TCM theory, RA belongs to the category of therapeutic concept “Bi Zheng ”, which is related to the deficiency of vital qi as well as the invasion of “wind (feng)”, “cold (han)”, “damp (shi)”, “stasis (yu)” and other evil qi [7, 8]. TCM can regulate the body function as a whole through a multifaceted approach and multiple mechanisms [9], therefore, it usually adopts the principles of warming the meridian, dispersing the cold and relieving the pain in RA treatment.

The compound Xuanju capsule, as a Chinese patent medicine, is composed of black ants (XJ), Herba Epimedii (*Epimedium brevicornu* Maxim., YYH), Fructus Cnidii (*Cnidium monnieri* (L.) Cuss., SCZ), and Fructus Lycii (*Lycium barbarum* L., GQZ). The black ants, as the primary drug, can nourish the kidney and liver, invigorate the circulation of blood and remove blood stasis, drive wind, and disperse cold [10]. Herba Epimedii, as the subject drug, can tonify the kidney, strengthen yang, and dispel wind and dampness [11]. Fructus Cnidii, as the adjuvant drug, can warm the kidney, invigorate yang, and dissipate cold [12]. Fructus Lycii, as the envoy drug, can tonify the liver and kidney and firm bones [13]. The combined use of four drugs has a good therapeutic effect on RA in clinical practice by warming and tonifying kidney-yang and removing wind and cold [14]. In addition, the compound Xuanju capsule could inhibit Th17 cells to secrete IL-17, while the secretion of Th17 and IL-17 played an important role in the pathogenesis of RA [15]. Furthermore, earlier researches have shown that the compound Xuanju capsule had the immunomodulatory and anti-inflammatory effects [16]. It had a significant inhibitory effect on the phagocytic function of the reticuloendothelial system and the delayed allergic reaction in mice and significantly inhibited the increase in the amount of abdominal exudate and the number of white blood cells induced by carboxymethyl cellulose stimulation. At the same time, it could also inhibit the formation of cotton ball granulation tissue. Modern pharmacological studies have also found that black ants contained rich formic acid and a variety of trace elements, had obvious anti-inflammatory effect, could reduce the tissue damage, and eliminate free radicals to promote bone formation [17]; icariin, the core component of Epimedii, could inhibit the inflammatory response and the synthesis of inflammatory substances and enhance the nonspecific immune function and cellular immunity [18]; osthole, a bioactive coumarin compound, was mainly contained in Fructus Cnidii and could significantly inhibit the release of inflammatory cytokines TNF-*α* and IL-6 and the inflammatory mediator NO [19]. However, the pharmacodynamic basis and more molecular mechanism of compound Xuanju capsule-treated RA are still unknown.

The network pharmacology method of TCM based on the analysis of network big data can effectively uncover the potential compatible action mechanism of drugs, thus providing a powerful tool for accelerating the modernization of TCM research and development [20]. In the present study, the method of TCM network pharmacology was adopted to further analyze the potential active ingredient, related targets and molecular signaling pathways of compound Xuanju capsule-related RA, with the aim of providing more theoretical basis for the effectiveness of compound Xuanju capsule-treated RA.

## 2. Methods

### 2.1. Screening of Active Ingredients of the Compound Xuanju Capsule

The active ingredients of black ants (XJ), Herba Epimedii (YYH), Fructus Lycii (SCZ) and Fructus Cnidii (GQZ) in the whole prescription were collected by the Traditional Chinese Medicine Systems Pharmacology (TCMSP) database and analysis platform (http://lsp.nwu.edu.cn/tcmsp.php), Traditional Chinese Medicine Integrated Database (TCMID, http://www.http://megabionet.org/tcmid), Natural Products Database of Traditional Chinese Medicine of Shanghai Institute of Organic Chemistry (http://www.chemcpd.csdb.cn) and the related literature of the compound Xuanju capsule [21, 22]. According to the integrative absorption, distribution, metabolism and excretion (ADME) model, bioavailability (OB) and drug-likeness (DL) were regarded as the screening criteria for active ingredients. The active ingredients were remained which were satisfactory with criteria of OB ≥ 30% and DL ≥ 0.18 for further study [23].

### 2.2. Prediction and Visualization of Active Ingredient-Potential Targets

To obtain the target of the each active ingredient in the compound Xuanju capsule, the potential targets were predicted from the TCMSP and STITCH databases (https://string-db.org). And then, the targets corresponding to the active ingredients screened from the two target databases were standardized in the UniProt database (http://www.uniprot.org), with the properties set to “reviewed” and “human” [24]. Subsequently, these standardized targets were merged and the duplicated items were removed. Finally, an active ingredient-target network was constructed using Cytoscape 3.7.1 software, and the network topology analysis was carried out. The degree, a topological parameter, was applied to screen the kernel active ingredients and the corresponding targets of the compound Xuanju capsule.

### 2.3. Prediction and Visualization of RA-Related Disease Targets

With “Rheumatoid arthritis” to be the keyword, RA-related targets were retrieved from three public online databases, namely, the Therapeutic Target Database (TTD, http://bidd.nus.edu.sg/group/cjttd), the PharmGKB database (https://www.Pharmgkb.org), the Online Mendelian Inheritance in Man (OMIM) database (http://www.omim.org) and the GeneCards database (https://www.genecards.org) [25, 26]. These targets were also sent to the UniProt database for normalization, and then, the duplicate targets were removed. Eventually, the RA-related disease target network was built by using Cytoscape 3.7.1 software as well.

### 2.4. Construction of the Protein-Protein Interaction (PPI) Network and Screening of the Common Targets

The active ingredient targets of the compound Xuanju capsule and the RA-related disease targets were analyzed using the STRING database, and “Homo sapiens” was limited. Two PPI networks of the active ingredient targets of the compound Xuanju capsule and the RA-related disease targets were built and merged through Cytoscape 3.7.1 software. Afterward, the common network (the intersection part of two PPI networks) was further analyzed by using Cytoscape 3.7.1 software, and the common targets were obtained. Additionally, we further screened targets whose degree value ranked in the top 20 as the core targets of compound Xuanju capsule-related RA.

### 2.5. Enrichment Analysis of the Common Targets

The Gene Ontology (GO) [27] biology process annotation and the Kyoto Encyclopedia of Genes and Genomes (KEGG) [28] pathway enrichment analysis of the common targets were carried out through the Metascape online database (http://metascape.org), which got the potential signal pathways, related biological processes, cell components, and molecular functions of the active components in the compound Xuanju capsule. The results of GO enrichment analysis and the bubble diagram of KEGG pathway enrichment were drawn using the Bioinformatics online tools (http://www.bioinformatics.com.cn). The ClueGO plug-in in Cytoscape 3.7.1 software was applied for constructing the visual results of the target proteins involved in signal pathways.

## 3. Results

### 3.1. Collection of Active Ingredients of the Compound Xuanju Capsule

In the present study, we searched all the reported chemical components in the compound Xuanju capsule through TCMSP, TCMID, and Natural Products Database of Traditional Chinese Medicine of Shanghai Institute of Organic Chemistry. A total of 478 ingredients were collected, among which 46 was found in black ants, 130 in *Epimedium*, 114 in Fructus Cnidii, and 188 in wolfberry. According to the screening criteria of the ADME, including the OB and DL, 87 active ingredients of the compound Xuanju capsule were obtained after duplicated targets were removed. In addition, three antirheumatic active ingredients of Xuanju were found from literatures, namely, citral, formic acid, and *β*-sitosterol [29]. Including all the above active ingredients, a total of 90 active ingredients of the compound Xuanju capsule were obtained.

### 3.2. Active Ingredient-Potential Targets and the Visualized Network

**Figure 1:**
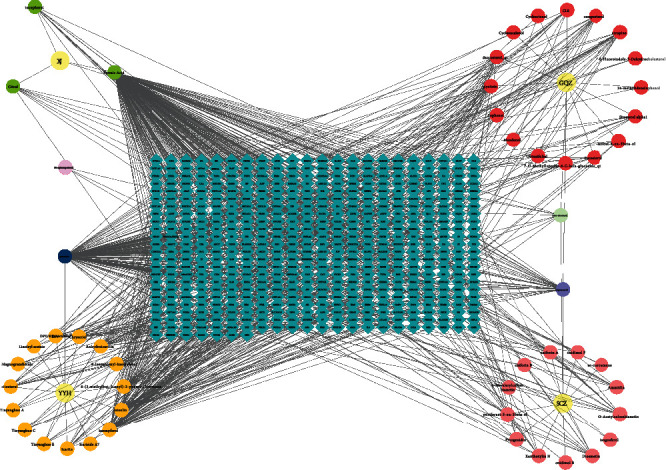
The active ingredient-target network of the compound Xuanju capsule. The blue diamonds depict the targets; yellow rounds delineate the herbs; green, orange, pink, and red rounds delineate XJ's, YYH's, SCZ's, and GQZ's active ingredients, respectively; and other rounds portray the common ingredients.

STITCH and TCMSP databases were used to predict the targets of the active ingredients. After the “Homo sapiens” targets were remained and the duplicated targets were removed, 51 active ingredients and 513 corresponding action targets were screened, as shown in Table 1. The above targets were imported into Cytoscape 3.7.1 software to construct the active ingredient-target network diagram (Figure 1). The network contained 570 important nodes and 936 edges. Among them, the formic acid, quercetin, kaempferol, luteolin, and stigmasterol had higher degree values, PTGS2, PTGS1, PRKACA, GABRA1 and RXRA targets had higher degree values. The top 10 active ingredients and the corresponding targets are shown in Table 2.

### 3.3. RA-Related Disease Targets and the Visualized Network

**Figure 2:**
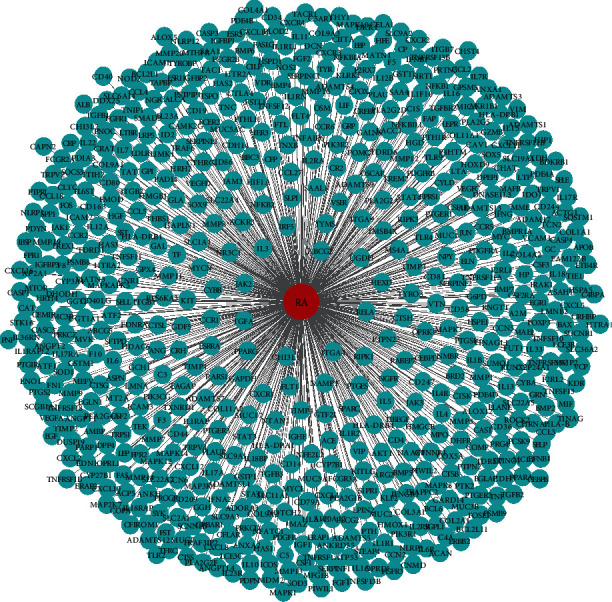
The RA-related disease-target network. The red round represents the disease (RA), and the blue rounds delineate targets related to RA.

Searching for RA-related targets through TTD, PharmGKB, OMIM and GeneCards databases, when “Homo sapiens” targets were remained and the duplicated targets were removed, a total of 641 targets were obtained. The above targets were submitted in the Cytoscape 3.7.1 software to construct the RA-related disease-target network (Figure 2).

### 3.4. PPI Network Topology Analysis

**Figure 3:**
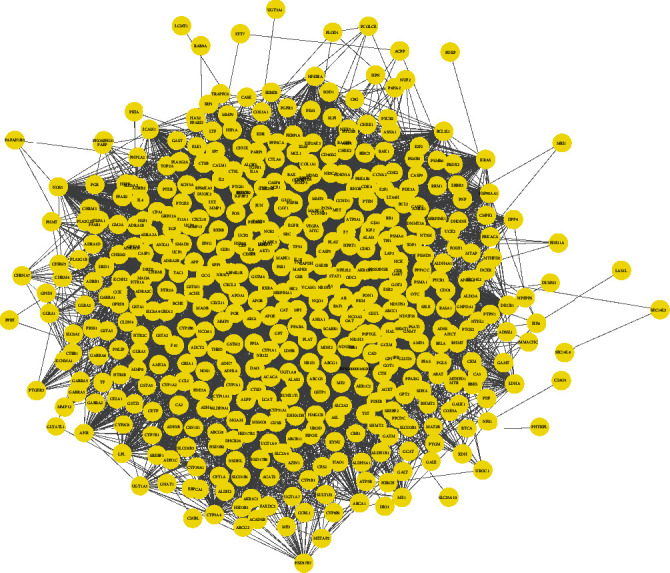
The PPI network of the active ingredient targets that consists of 500 nodes and 9488 edges. The yellow rounds represent the protein targets acted by the active ingredient, and the black lines denote the interaction relationship between the protein targets.

**Figure 4:**
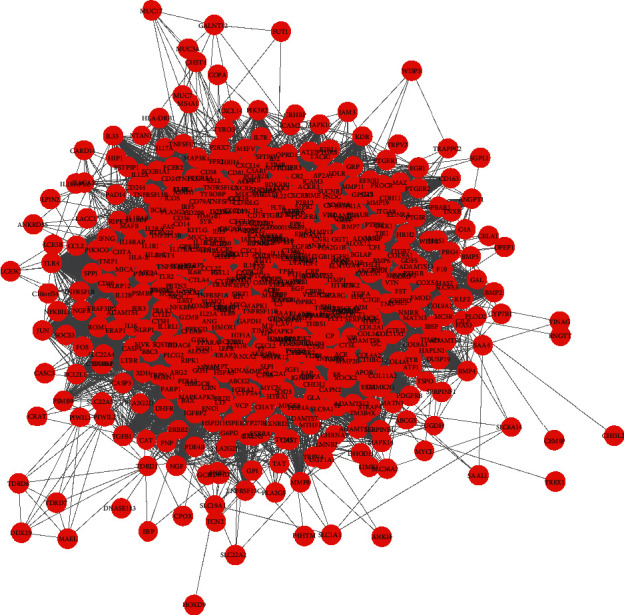
The PPI network of RA-related disease targets that consists of 625 nodes and 19389 edges. The red nodes represent the protein targets worked on the RA, and the black lines denote the interaction relationship between the protein targets.

The STRING online database was used for PPI network topology analysis. The analyzed PPI relationship data results (.tsv format file) were imported into the Cytoscape 3.7.1 software, and then, the visualized PPI network of the active ingredient targets (Figure 3) and the PPI network of RA-related disease targets (Figure 4) were obtained. Among them, the PPI network of the active ingredient targets involved 500 nodes (interaction proteins) and 9488 edges (interaction relationships); the PPI network of RA-related disease targets involved a total of 625 nodes (interaction proteins) and 19389 edges (interaction relationships).

### 3.5. Screening of the Common Targets

**Figure 5:**
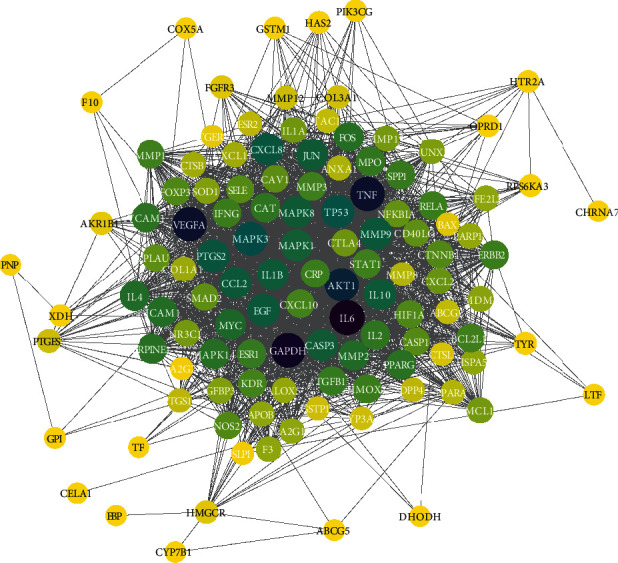
The common target PPI network that consists of 116 nodes and 2241 edges. The 116 rounds represent the potential protein targets of compound Xuanju capsules against RA, and the black lines denote the interaction relationship between the protein targets. The color and size of the round indicate the size of the degree value; the degree value corresponds to the round color from yellow to purple.

In the Cytoscape 3.7.1 software, the CytoNCA plug-in was applied to merge two PPI networks and obtain a visualized intersection target network (Figure 5), which consisted of 116 common nodes and 2241 edges. As shown in Figure 5, these key targets were highly correlated in the potential target network relationship of compound Xuanju capsule-treated RA, including IL-6, VEGFA, TNF, AKT1, MAPK3, CXCL8, IL10, IL1*β*, JUN and CASP3. The 116 common targets were ranked according to the degree value, and the top 20 core targets of compound Xuanju capsule-related RA are shown in Table 3.

### 3.6. GO Enrichment Analysis

**Figure 6:**
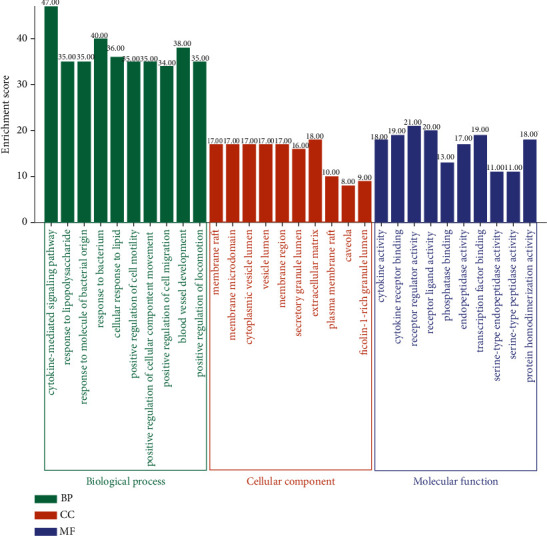
GO enrichment analysis of the common targets. The top 10 GO functional terms were selected (*P* < 0.05). BP: biological processes; CC: cellular components; MF: molecular functions.

GO enrichment analysis includes molecular function (GO-MF), biological process (GO-BP), and cellular component (GO-CC). The top 10 GO analysis results were selected according to the degree of importance and significance, as shown in Figure 6. The GO-MF analysis indicated that the core targets of compound Xuanju capsule-related RA mainly affected the cytokine activity, the receptor regulator activity, the receptor ligand activity, cytokine receptor binding, phosphatase binding, transcription factor binding, and other molecular functions in the treatment of RA. The GO-BP analysis showed that the core targets of compound Xuanju capsule-related RA could be involved in a majority of biological processes in the treatment of RA, such as the cytokine-mediated signaling pathways, the response to lipopolysaccharide, the response to molecule of bacterial origin, the response to bacterium, the cellular response to lipid, the positive regulation of cell motility, the positive regulation of cellular component movement, the positive regulation of cell migration, the blood vessel development, and the positive regulation of locomotion. The GO-CC analysis indicated that the core targets of compound Xuanju capsule-related RA were primarily enriched in the membrane rafts, the membrane microdomain, the cytoplasmic vesicle lumen, the secretory granule lumen, the extracellular matrix and so on.

### 3.7. KEGG Pathway Analysis

**Figure 7:**
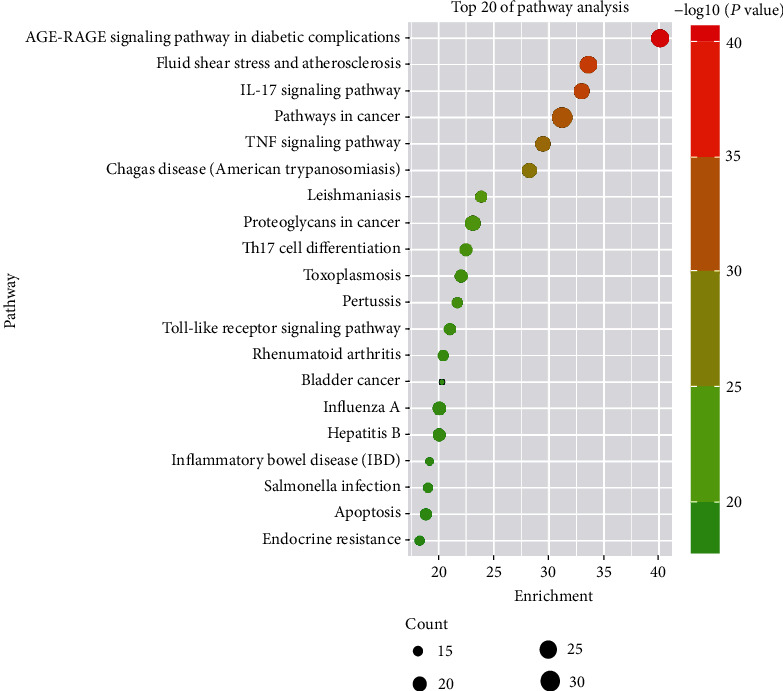
KEGG pathway enrichment analysis of the common targets. The top 20 pathways were identified. The color represented *P* value, and the size of the spot represented count of genes.

**Figure 8:**
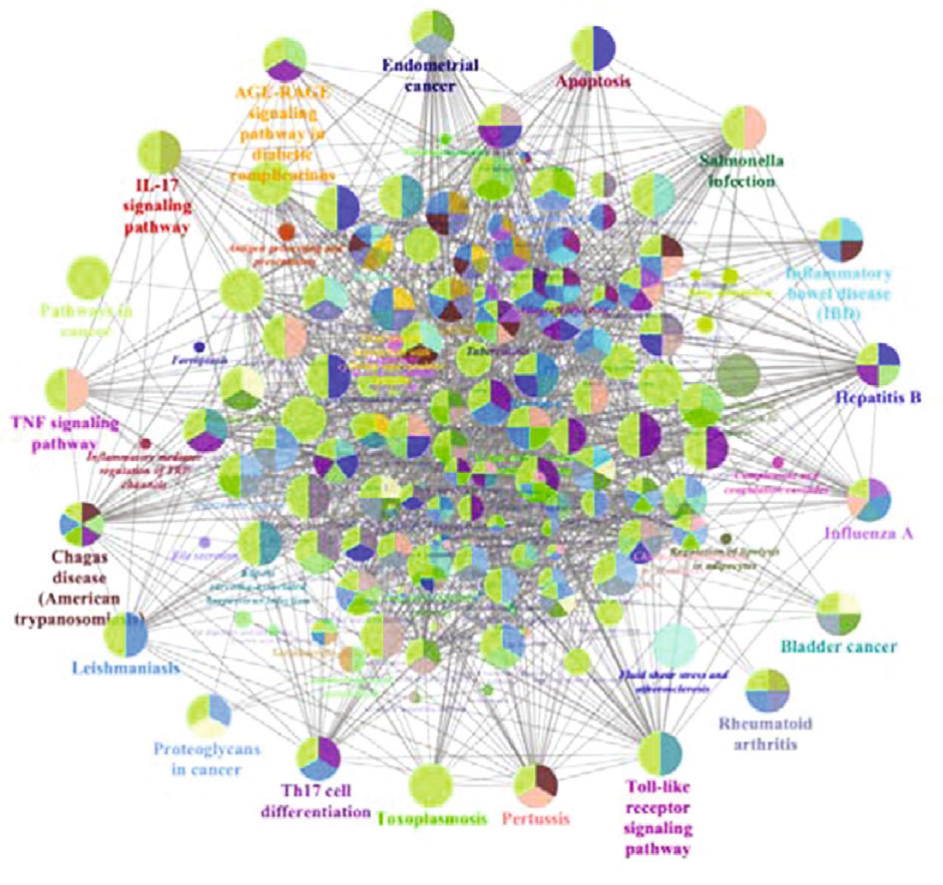
KEGG signal pathway enrichment analysis of core targets in the compound Xuanju capsule treating RA. KEGG terms are represented as nodes, and the size of the node indicates the size of the degree value; the degree value corresponds to the node size from small to big.

The KEGG pathway analysis was carried out to further explore which pathways were related to the screened common targets. Among the top 20 pathways (sorted by the degree value), there were mainly the IL-17 signaling pathway, the TNF signaling pathway, the Th17 cell differentiation pathway, the Toll receptor signaling pathway, the RA pathway, the fluid shear stress and atherosclerosis pathway, and so on (Figure 7). Using the ClueGO plug-in in Cytoscape 3.7.1 software, the visualization results of the signal pathway of the target proteins involved were constructed. It was found that the efficacy of the compound Xuanju capsule was mainly achieved by regulating the IL-17 signaling pathway, the TNF signaling pathway, the Th17 cell differentiation pathway, the Toll receptor signaling pathway, and so on, as shown in Figure 8.

## 4. Discussion

Network pharmacology is a research method to clarify the disease occurrence and development from the perspectives of systems biology and biological networks, through the collection of ingredients and action targets; an interaction network is constructed to further investigate the regulation effect of TCM on different signals, thereby revealing the action mechanism of TCM [30]. Based on the network pharmacology tactics, this study deeply explored the potential mechanism of the Chinese patent medicine compound Xuanju capsule in the treatment of RA. 51 active ingredients of compound Xuanju capsules, such as formic acid, quercetin, kaempferol, luteolin and stigmasterol, were screened through multiple large databases. It was predicted that there were 116 potential targets of the compound Xuanju capsule in the treatment of RA, such as IL-6, GAPDH, VEGFA, TNF and AKT1. Five potential mechanism pathways of compound Xuanju capsule-treated RA were analyzed and summarized, namely, the IL-17 signaling pathway, the TNF signaling pathway, the Th17 cell differentiation pathway, the Toll receptor signaling pathway and the RA pathway.

Among the 51 active ingredients selected in this work, formic acid, quercetin, kaempferol, luteolin, stigmasterol, anhydroicaritin and so on were the top 10 active ingredients according to the degree value, which might be the main active ingredients of the compound Xuanju capsule against RA. Researches have demonstrated that formic acid as one of the biologically active substances of black ants was the material basis for its anti-inflammatory, analgesic, antibacterial and antirheumatic effects and immune regulation and prevention of arteriosclerosis [17, 31]. Quercetin can improve morning stiffness, arthralgia, and other clinical symptoms of RA patients and reduce disease activity scores [32]. Kaempferol can inhibit the migration and invasion of fibroblast-like synovial cells in RA by blocking the activation of the MAPK pathway and inhibit the reorganization of the actin cytoskeleton during cell migration, thereby reducing the arthritis symptoms of collagen-induced arthritis rats [33]. Luteolin can reduce the proliferation of fibroblast-like synovial cells in RA by inhibiting the NF-*κ*B and JAK/STAT signal pathways [34]. Stigmasterol is a natural plant product with anti-inflammatory activity, which can inhibit the signal pathway of the inflamed joints, improve the antioxidant status, and transfer inflammation [35]. Anhydroicaritin has the function of repairing the bone tissue; in addition, it can promote the angiogenesis by increasing the expression of proangiogenic factors such as VEGFA and McP-1, thus providing more nutrients for bone formation [36]. Therefore, the above-mentioned active ingredients might be all the medicinal material bases of the compound Xuanju capsule in the treatment of RA, which had an important reference value for subsequent clinical and experimental research.

Each node in the network represents the protein corresponding to the action target; thus, 116 common nodes involved in the intersection network indicated that there were a total of 116 potential action targets of the compound Xuanju capsule in the treatment of RA. Additionally, the size and color of the nodes represent the degree value of the target protein, and the connection between the nodes represents the potential interaction between the target proteins [37]. The target protein in the network relationship is greatly correlated with the node size, the node color, and the connections, which increase with them [38]. Hence, IL-6, VEGFA, TNF, AKT1, MAPK3, CXCL8, IL10, IL1*β*, JUN, CASP3 and other targets showed an obvious correlation in the network relationship of compound Xuanju capsules in the treatment of RA, as shown in Figure 5. This demonstrated that the compound Xuanju capsule had higher binding activity with these targets which could be used as the potential action targets. It had shown that TNF-*α* was increased in the synovium and cartilage of RA patients, which could promote the cartilage destruction by inhibiting the collagen synthesis, stimulating the fibroblasts and chondrocytes to produce the prostaglandins and collagenase, stimulating the chondrocytes to secrete metalloproteinases, inducing the differentiation of peripheral blood mononuclear cells into osteoclasts, etc. [39]. Meanwhile, TNF-*α* can increase the production of the proinflammatory factors (IL1*β*, IL-6, etc.) and chemokines (CXCL8, etc.) through the cascade reaction and further aggravate the inflammatory response [40]. IL-6 is a major inflammatory mediator and induced osteoclast precursors to differentiate into the real osteoclasts during bone metabolism [41]. Thus, IL-6 is the main factor for further destruction of bone and cartilage. As the negative regulator of the proinflammatory cytokines, 1L-10 can reduce the release of the proinflammatory cytokines [42]. One of the earliest observed phenomena in synovitis is the formation of new blood vessels. VEGF is the key molecule involved in the regulation of angiogenesis which can promote the infiltration of inflammatory cells into the joint and lead to synovial hyperplasia and progressive bone destruction [43, 44]. AKT1 is believed to be closely related to the production of RA synovial fibroblasts [45]. The MAPK gene participates in signaling pathways that can reduce the oxygen free radicals, inhibit the cell proliferation, promote the apoptosis, reduce the inflammatory response, and inhibit the angiogenesis, thus effectively inhibiting RA progression [46]. JUN, closely associated with the proliferation, differentiation, maturation, and function of the osteoblast, plays an important role in maintaining the dynamic balance of bone formation and bone resorption [38]. The results of the current study showed that the known active ingredients of the compound Xuanju capsule could primarily act on 116 targets in the treatment of RA. Among the 116 targets, IL-6, VEGFA, TNF, AKT1, MAPK3, CXCL8, IL10, IL1*β*, MAPK8, JUN and other targets had higher degree values, indicating that these targets might be the core targets of the compound Xuanju capsule against RA, and the corresponding action mechanism was characterized by multiple components and multiple targets.

The GO and KEGG pathway enrichment analysis were conducted on 116 common targets. The GO results found that the cytokine activity and the cytokine receptor binding were closely related to the occurrence of RA in terms of molecular functions. In addition, the signal pathways and vascular development mediated by the cytokines in biological processes had a certain impact on RA; this was also corroborated with the results that the compound Xuanju capsule might act on 166 potential core targets in the treatment of RA. KEGG pathway enrichment analysis results demonstrated that the main signaling pathways regulated by the compound Xuanju capsule were the IL-17 signaling pathway, the TNF signaling pathway, the Th17 cell differentiation pathway, the Toll receptor signaling pathway, the RA pathway, the liquid shear stress and atherosclerosis pathways, etc. It had shown that the TNF signaling pathway was the key pathway derived from the enrichment analysis and an important pathway in the inflammatory response [47]. The occurrence and development of RA are related to the abnormal expression of IL-6, IL1*β*, TNF and PTGS2 in the TNF signaling pathway [48]. IL-17 not only causes the inflammation but also stimulates the osteoclast differentiation to cause bone and cartilage damage. At the same time, IL-17 can also stimulate the production of many types of chemokines and aggravate the inflammatory response. Furthermore, IL-17 can induce the prostaglandins to enter the inflammatory sites through the cyclooxygenase-2 and then damage the synovial surface and articular cartilage [49]. Therefore, the IL-17 signaling pathway plays an important role in the course of RA. The RA pathway includes the T cell receptor signaling pathway, the Toll receptor signaling pathway, the VEGF signaling pathway, the osteoclast differentiation, and the Th17 cell differentiation, all of which are related to the immune regulation and inflammation. Among them, the abnormal T cell-mediated immune response is the main pathogenesis of RA. In the diseased synovial tissue, a large number of activated T lymphocytes gather around the blood vessels. The activated T lymphocytes interact directly with synovial macrophages, secrete the cytokines, and stimulate the synovial cells to release collagenase and protease, which further caused the cartilage and bone damage [38]. The Toll receptor signaling pathway directly triggers the intracellular bactericidal mechanism or induces the production of immune inflammatory factors on the basis of identifying pathogenic microorganisms to expand nonspecific defenses. Research manifested that the Toll receptors were significantly expressed in the pathological tissues of RA patients [50]. The VEGF signaling pathway inhibits the expression of cytokines in arthritis, reduces the production of collagen, inhibits the proliferation of synovial cells, and weakens the invasion ability [41]. Th17 and Treg cells are two subtypes of CD4^+^ T cells. Among them, Th17 cells can highly secrete inflammatory cytokines such as IL-17, IL-6 and IL-22, mediating inflammatory reactions and autoimmune reactions in the body [51]. Abnormal activation of Th17 cells will cause the imbalance of Th17/Treg cells and play a key role in autoimmune diseases such as RA, especially in local inflammatory response and bone destruction, while long-term local inflammation will induce and worsen the destruction of RA articular cartilage [52]. Cells in the body can not only sense the stimulation of various chemical factors in the external environment but also live in various mechanical environments, such as the pressure of fluid as well as shear stress caused by the fluid flow. In the interaction between the extracellular matrix and the signal molecules on the cell membrane, these mechanical stresses can be sensed by cells, thereby activating intracellular signaling pathways, transforming the mechanical signals into the intracellular biochemical signals, and thus affecting cell shape, adhesion and migration, gene expression, differentiation, and even cell death [53]. These mechanical factors play a decisive role in the changes of cellular functions such as cardiovascular disease, bone formation, and organ development. The present study results indicated that the main signaling pathways regulated by the compound Xuanju capsule included the IL-17 signaling pathway, the TNF signaling pathway, the Toll receptor signaling pathway, the Th17 cell differentiation pathway, the RA pathway and the liquid shear stress and atherosclerosis pathway. Furthermore, the inflammatory signal pathways were the main pathway. This suggested that the compound Xuanju capsule might mainly regulate the inflammatory pathways to protect the bone tissue and improve the clinical symptoms of RA patients such as joint swelling and pain. Moreover, the above research results predicted by the network pharmacology method were mutually confirmed with the observation results of the compound Xuanju capsule in the clinical treatment of RA [14, 15].

RA is a chronic inflammatory autoimmune disease with the complex pathogenesis [35]. The results of the present study suggested that RA was related to the abnormality of the IL-6-based multitarget and IL-17 pathway-based multisignal pathways; thus, the pathogenesis of RA had the characteristics of multitarget and multipathway. To some extent, it explained the phenomenon that there was no good effect in the single target treatment of the disease in clinic. TCM has the characteristics and advantages of multicomponent, multitarget, and multipathway in the treatment of diseases. Therefore, it is still a research hotspot to find drugs for RA treatment.

In conclusion, based on the network pharmacology methods, the current study found that the compound Xuanju capsule, in the treatment of RA, might mainly act on 116 targets based on IL-6 through 51 active ingredients such as formic acid, quercetin and kaempferol, and then regulated the IL-17 signal pathway, the TNF signal pathway, the Th17 cell differentiation pathway, the Toll receptor signaling pathway, the RA pathway, etc., to further affect the release of the proinflammatory factors and the anti-inflammatory factors, thereby alleviating inflammatory damage and improving bone tissue repair. The above results reflected the mechanism and advantages of the compound Xuanju capsule in treating RA with multiple components, multiple targets, and multiple pathways, and also confirmed that inhibiting the secretion of inflammatory factors was one of its major mechanisms.

## Figures and Tables

**Table 1:** 51 active ingredients and the number of the corresponding potential targets of the compound Xuanju capsule.

Chemical component	Target number	OB (%)	DL
Tocopherol	10	32.29	0.70
Citral	8	22.52	0.52
Formic acid	236	33.26	0.00
Beta-sitosterol	7	15.00	0.81
24-Epicampesterol	2	37.58	0.71
Linoleyl acetate	3	42.10	0.20
Sitosterol	11	36.91	0.75
DFV	10	32.76	0.18
Chryseriol	14	35.85	0.27
8-Isopentenyl-kaempferol	12	38.04	0.39
Kaempferol	65	41.88	0.24
Anhydroicaritin	28	45.41	0.44
Yinyanghuo A	4	56.96	0.77
Yinyanghuo C	5	45.67	0.50
Yinyanghuo E	4	51.63	0.55
8-(3-Methylbut-2-enyl)-2-phenyl-chromone	24	48.54	0.25
Icariin	9	41.58	0.61
Icariside A7	1	31.91	0.86
Luteolin	52	36.16	0.25
Magnograndiolide	4	63.71	0.19
Quercetin	153	46.43	0.28
Poriferast-5-en-3beta-ol	7	36.91	0.75
Ammidin	8	34.55	0.22
Diosmetin	10	31.14	0.27
Xanthoxylin N	8	35.51	0.21
Prangenidin	6	36.31	0.22
Ar-curcumene	2	52.34	0.65
Cnidimol B	1	68.66	0.26
Cnidimol F	3	54.43	0.28
Cniforin A	5	55.89	0.47
Cniforin B	1	36.70	0.60
O-Acetylcolumbianetin	11	60.04	0.26
Isogosferol	1	30.07	0.25
o-Isovalerylcolum bianetin	6	64.03	0.36
Stigmasterol	34	43.83	0.76
Sitosterol alpha1	7	43.28	0.78
Cycloartenol	2	38.69	0.78
Mandenol	2	42.00	0.19
Atropine	18	45.97	0.19
Campesterol	12	37.58	0.71
24-Methylidenelophenol	1	44.19	0.75
Daucosterol	16	36.91	0.75
Glycitein	23	50.48	0.24
CLR	9	37.87	0.68
Fucosterol	7	43.78	0.76
Lophenol	6	38.13	0.71
6-Fluoroindole-7-dehydrocholesterol	2	43.73	0.72
7-O-Methylluteolin-6-C-beta-glucoside	3	40.77	0.30
Cycloeucalenol	1	39.73	0.79
Lanost-8-en-3beta-ol	4	34.23	0.74
Obtusifoliol	2	42.55	0.76

**Table 2:** The top 10 active ingredients and the corresponding target degree values of the compound Xuanju capsule.

Chemical component	Degree	Component target	Degree
Formic acid	237	PTGS2	12
Quercetin	155	PTGS1	10
Kaempferol	66	PRKACA	10
Luteolin	53	GABRA1	9
Stigmasterol	36	RXRA	8
Anhydroicaritin	29	AR	8
8-(3-Methylbut-2-enyl)-2-phenyl-chromone	25	PRSS1	8
Glycitein	24	SCN5A	8
Atropine	19	NCOA2	8
Daucosterol	17	ESR1	8

**Table 3:** The information of top 20 common targets.

Gene	Protein	UniProt ID	Degree	Betweenness centrality	Closeness centrality	Neighborhood connectivity
IL6	Interleukin-6	P05231	93	667.9930402	0.833333333	89.12839505
GAPDH	Glyceraldehyde-3-phosphate dehydrogenase	P04406	91	719.0841193	0.815602837	85.92631575
VEGFA	Vascular endothelial growth factor A	P15692	89	413.5753955	0.793103448	84.52425102
TNF	Tumor necrosis factor	P01375	89	335.4934403	0.798611111	84.68661002
AKT1	RAC-alpha serine/threonine-protein kinase	P31749	87	524.2608782	0.798611111	80.85548315
MAPK3	Mitogen-activated protein kinase 3	P27361	84	410.4623204	0.777027027	76.40073952
TP53	Cellular tumor antigen p53	P04637	81	307.2138965	0.746753247	73.70696197
CXCL8	Interleukin-8	P10145	78	218.2893964	0.732484076	70.86636431
PTGS2	Prostaglandin G/H synthase 2	P35354	78	206.0168462	0.737179487	69.98848441
EGF	Proepidermal growth factor	P01133	76	261.8569934	0.737179487	65.03746714
MMP9	Matrix metalloproteinase-9	P14780	76	433.8806129	0.71875	68.81580781
CCL2	C-C motif chemokine 2	P13500	75	157.4214188	0.72327044	67.00969048
IL10	Interleukin-10	P22301	74	145.584267	0.71875	65.80203946
IL1*β*	Interleukin-1 beta	P01584	74	158.014336	0.71875	65.60821648
MAPK8	Mitogen-activated protein kinase 8	P45983	73	129.355471	0.709876543	65.62343779
JUN	Transcription factor AP-1	P05412	73	122.1960842	0.709876543	66.15527831
CASP3	Caspase-3	P42574	72	107.386397	0.701219512	64.73650484
MAPK1	Mitogen-activated protein kinase 1	P28482	71	166.1161984	0.705521472	61.56264833
MYC	Myc proto-oncogene protein	P01106	64	147.9851483	0.67251462	54.86995265
MMP2	72 kDa type IV collagenase	P08253	62	74.82792554	0.66091954	52.92741783

## Data Availability

We have presented all our main data in the form of figures and tables. The datasets supporting the conclusions of this article are included within the article.

## References

[1] Sparks J. A. (2019). Rheumatoid arthritis. *Annals of Internal Medicine*.

[2] Wang W., Wang X., Tang X., Jiang Q., Fan Y. (2019). Classifying rheumatoid arthritis by traditional Chinese medicine Zheng: a multi-center cross-sectional study. *Journal of Traditional Chinese Medicine*.

[3] Pisetsky D. S. (2017). Advances in the treatment of rheumatoid arthritis: costs and challenges. *North Carolina Medical Journal*.

[4] Giannini D., Antonucci M., Petrelli F., Bilia S., Alunno A., Puxeddu I. (2020). One year in review 2020: pathogenesis of rheumatoid arthritis. *Clinical and Experimental Rheumatology*.

[5] Gou K., Zeng R., Ren X. (2018). Anti-rheumatoid arthritis effects in adjuvant-induced arthritis in rats and molecular docking studies of *Polygonum orientale* L. extracts. *Immunology Letters*.

[6] Zhang L., Cao Z., Yang Y., Tan X., Mao J., Su L. (2020). Traditional Chinese medicine on treating active rheumatoid arthritis: a protocol for systematic review and meta-analysis. *Medicine*.

[7] Lu S., Wang Q., Li G., Sun S., Guo Y., Kuang H. (2015). The treatment of rheumatoid arthritis using Chinese medicinal plants: from pharmacology to potential molecular mechanisms. *Journal of Ethnopharmacology*.

[8] Li X., Zhang S. (2020). Herbal compounds for rheumatoid arthritis: literatures review and cheminformatics prediction. *Phytotherapy Research*.

[9] Wang H., Wang M. (2019). Nanometer preparation of traditional Chinese medicine for rheumatoid arthritis. *Zhongguo Zhong Yao Za Zhi*.

[10] Wang Q., Zhou C., Xiao Y., Wu Z., Wei W., Cai J. (2020). Effects of Xuanju extract on benign prostatic hyperplasia. *Chinese Journal of Andrology*.

[11] Wang L., Li Y., Guo Y. (2016). Herba Epimedii: an ancient Chinese herbal medicine in the prevention and treatment of osteoporosis. *Current Pharmaceutical Design*.

[12] Zhang W., Ma D., Zhao Q., Ishida T. (2010). The Effect of the Major Components of Fructus Cnidii on Osteoblasts *In Vitro*. *Journal of Acupuncture and Meridian Studies*.

[13] Lu K.-H., Liu C.-T., Raghu R., Sheen L.-Y. (2012). Therapeutic potential of Chinese herbal medicines in alcoholic liver disease. *Journal of Traditional and Complementary Medicine*.

[14] Wang L., Wang C., Wang X., Zhang Y. (2018). Clinical study of compound Xuanju capsule combined with methotrexate in the treatment of refractory rheumatoid arthritis. *Chinese Traditional Patent Medicine*.

[15] Wang C., Wang X., Zhang Y., Wang L. (2016). Effect of compound Xuanju capsules on Th17 cells/interleukin 17 in patients with rheumatoid arthritis. *Pharmacology and Clinics of Chinese Materia Médica*.

[16] Jia W., Xue J., Wang Y., Huo H. (2003). Study on immunoregulatory and anti-inflammatory effects of compound Xuanju capsule. *Chinese Traditional and Herbal Drugs*.

[17] He Y., Li G. (2003). New progress in pharmacological research of ant preparation. *Lishizhen Medicine and Materia Medica Research*.

[18] Wang Z., Wang D., Yang D., Zhen W., Zhang J., Peng S. (2018). The effect of icariin on bone metabolism and its potential clinical application. *Osteoporosis International*.

[19] Kong L., Yao Y., Xia Y., Liang X., Ni Y., Yang J. (2019). Osthole alleviates inflammation by down-regulating NF-kappa B signaling pathway in traumatic brain injury. *Immunopharmacology and Immunotoxicology*.

[20] Tao Q., du J., Li X. (2020). Network pharmacology and molecular docking analysis on molecular targets and mechanisms of Huashi Baidu formula in the treatment of COVID-19. *Drug Development and Industrial Pharmacy*.

[21] Jin Q., Lu J., Gao R., Xu J., Pan X., Wang L. (2021). Systematically deciphering the pharmacological mechanism of Fructus Aurantii via network pharmacology. *Evidence-Based Complementary and Alternative Medicine*.

[22] Qian C., Chen P., Zheng W. (2020). To explore the potential mechanism of Shanhaidan granules in the treatment of erectile dysfunction based on network pharmacology. *Chinese Pharmacological Bulletin*.

[23] Kim S. K., Lee S., Lee M. K., Lee S. (2019). A systems pharmacology approach to investigate the mechanism of Oryeong-san formula for the treatment of hypertension. *Journal of Ethnopharmacology*.

[24] Zhong P., Song L., Gao M. (2020). Network pharmacology-based strategy for predicting active ingredients and potential targets of Gegen Qinlian decoction for rotavirus enteritis. *Evidence-Based Complementary and Alternative Medicine*.

[25] Shi Y.-Y., Li Y.-Q., Xie X. (2020). Homotherapy for heteropathy active components and mechanisms of Qiang-Huo- Sheng-Shi decoction for treatment of rheumatoid arthritis and osteoarthritis. *Computational Biology and Chemistry*.

[26] Qian H., Jin Q., Liu Y. (2020). Study on the multitarget mechanism of Sanmiao pill on gouty arthritis based on network pharmacology. *Evidence-Based Complementary and Alternative Medicine*.

[27] Ashburner M., Ball C., Blake J. (2000). *Gene Ontology: tool for the unification of biology*. *Genetics*.

[28] Tanabe M., Kanehisa M. (2012). Using the KEGG database resource. *Current Protocols in Bioinformatics*.

[29] Wu Z., Wu B. (1995). Prospect of treatment of chronic diseases by Polyrhachis vicina Roger. *Bulletin of Biology*.

[30] Li H., Lv T., Wang B. (2021). Integrating network pharmacology and experimental models to investigate the mechanism of Huanglian Jiedu decoction on inflammatory injury induced by cerebral ischemia. *Evidence-Based Complementary and Alternative Medicine*.

[31] Song G. (2005). Medicinal use and nutrition of ants. *Food and Drug*.

[32] Kim H.-R., Kim B.-M., Won J. Y. (2019). Quercetin, a plant polyphenol, has potential for the prevention of bone destruction in rheumatoid arthritis. *Journal of Medicinal Food*.

[33] Pan D., Li N., Liu Y. (2018). Kaempferol inhibits the migration and invasion of rheumatoid arthritis fibroblast-like synoviocytes by blocking activation of the MAPK pathway. *International Immunopharmacology*.

[34] Lou L., Liu Y., Zhou J. (2015). Chlorogenic acid and luteolin synergistically inhibit the proliferation of interleukin-1*β*-induced fibroblast-like synoviocytes through regulating the activation of NF-*κ*B and JAK/STAT-signaling pathways. *Immunopharmacology and Immunotoxicology*.

[35] Ahmad Khan M., Sarwar A., Rahat R., Ahmed R., Umar S. (2020). Stigmasterol protects rats from collagen induced arthritis by inhibiting proinflammatory cytokines. *International Immunopharmacology*.

[36] Lai X., Huang X., Zeng Y. (2013). Protective effect of anhydroicaritin against peritonitis in mice. *Chinese Journal of Cellular and molecular immunology*.

[37] Reng S., Ni L., Meng D. (2019). Mechanisms of Cortex phellodendri-Herba tuberculate speranskia in treatment of rheumatoid arthritis based on network pharmacology. *Chinese Journal of New Drugs and Clinical Remedies*.

[38] Liang Q., Yan J., Feng Y., Li F., Xie T., Fang X. (2019). Effect and mechanism of YAO medicine compound containing Cissus pteroclada on rheumatoid arthritis in rats and its Q-marker prediction. *Chinese Traditional and Herbal Drugs*.

[39] Shi H., Wang D., Wu R., Li H. (2012). Role of tumor necrosis factor-*α* mediated nuclear factor kappa B signaling pathway in antiogenesis in rheumatoid arthritis. *Medical Recapitulate*.

[40] Morita T., Shima Y., Fujimoto K. (2019). Anti-receptor activator of nuclear factor *κ*B ligand antibody treatment increases osteoclastogenesis-promoting IL-8 in patients with rheumatoid arthritis. *International Immunology*.

[41] Ding W., Han L., Qian K., Lin C. (2020). Mechanism of Caulis Sinomenii on rheumatoid arthritis based on network pharmacology. *China Medical Herald*.

[42] Saxena A., Khosraviani S., Noel S., Mohan D., Donner T., Hamad A. R. A. (2015). Interleukin-10 paradox: a potent immunoregulatory cytokine that has been difficult to harness for immunotherapy. *Cytokine*.

[43] Zhang Y., Qiu H., Zhang H., Wang L., Zhuang C., Liu R. (2013). Vascular endothelial growth factor A (VEGFA) polymorphisms in Chinese patients with rheumatoid arthritis. *Scandinavian Journal of Rheumatology*.

[44] Elshabrawy H. A., Chen Z., Volin M. V., Ravella S., Virupannavar S., Shahrara S. (2015). The pathogenic role of angiogenesis in rheumatoid arthritis. *Angiogenesis*.

[45] Xie X., Wang Y., Luo S., Zhao Z., Li X. (2019). Pathogenesis of rheumatoid arthritis. *World Latest Medicine Information*.

[46] Shi L., Sun W. (2020). Mechanism of Shaoyao Gancao decoction in treatment of rheumatoid arthritis based on network pharmacology. *Chinese Herbal Medicines*.

[47] Mo W., Su Z., Liang Y. (2020). Study on the mechanism of Millettia speciosa Champ. in the treatment of rheumatoid arthritis based on network pharmacology. *Journal of Guangxi Medical University*.

[48] Osta B., Benedetti G., Miossec P. (2014). Classical and paradoxical effects of TNF-*α* on bone homeostasis. *Frontiers in Immunology*.

[49] Kirkham B. W., Kavanaugh A., Reich K. (2014). Interleukin-17A: a unique pathway in immune-mediated diseases: psoriasis, psoriatic arthritis and rheumatoid arthritis. *Immunology*.

[50] Yao R., Wang Y., Cai H. (2015). Research progress on the correlation between Toll-like receptor and rheumatoid arthritis. *Chinese Journal of Immunology*.

[51] Wang T., Sun X., Zhao J. (2015). Regulatory T cells in rheumatoid arthritis showed increased plasticity toward Th17 but retained suppressive function in peripheral blood. *Annals of the Rheumatic Diseases*.

[52] Paradowska-Gorycka A., Wajda A., Romanowska-Próchnicka K. (2020). Th17/Treg-related transcriptional factor expression and cytokine profile in patients with rheumatoid arthritis. *Frontiers in Immunology*.

[53] Chien S. (2006). Molecular basis of rheological modulation of endothelial functions: importance of stress direction. *Biorheology*.

